# Emerging Alzheimer’s disease therapeutics: promising insights from lipid metabolism and microglia-focused interventions

**DOI:** 10.3389/fnagi.2023.1259012

**Published:** 2023-10-25

**Authors:** Nour S. Tobeh, Kimberley D. Bruce

**Affiliations:** ^1^Division of Endocrinology, Metabolism and Diabetes, Department of Medicine, University of Colorado Anschutz Medical Campus, Aurora, CO, United States; ^2^Skaggs School of Pharmacy and Pharmaceutical Sciences, University of Colorado Anschutz Medical Campus, Aurora, CO, United States

**Keywords:** Alzheimer’s disease, microglia, lipids, amyloid, therapeutics

## Abstract

More than 55 million people suffer from dementia, with this number projected to double every 20 years. In the United States, 1 in 3 aged individuals dies from Alzheimer’s disease (AD) or another type of dementia and AD kills more individuals than breast cancer and prostate cancer combined. AD is a complex and multifactorial disease involving amyloid plaque and neurofibrillary tangle formation, glial cell dysfunction, and lipid droplet accumulation (among other pathologies), ultimately leading to neurodegeneration and neuronal death. Unfortunately, the current FDA-approved therapeutics do not reverse nor halt AD. While recently approved amyloid-targeting antibodies can slow AD progression to improve outcomes for some patients, they are associated with adverse side effects, may have a narrow therapeutic window, and are expensive. In this review, we evaluate current and emerging AD therapeutics in preclinical and clinical development and provide insight into emerging strategies that target brain lipid metabolism and microglial function – an approach that may synergistically target multiple mechanisms that drive AD neuropathogenesis. Overall, we evaluate whether these disease-modifying emerging therapeutics hold promise as interventions that may be able to reverse or halt AD progression.

## 1. Introduction

Alzheimer’s disease (AD) is the most common type of dementia and is characterized by progressive, and ultimately fatal, neurodegeneration (Breijyeh and Karaman, 2020). Currently, around 6.7 million Americans are living with the disease and this number is expected to increase to almost 13 million by 2050 (Alzheimer’s Association, 2021, 2023a,b). At least 55 million individuals are suffering from the disease worldwide and this number is expected to increase to 139 million by 2050 (BrightFocus Foundation, 2019). Furthermore, AD kills more individuals than breast cancer and prostate cancer combined (Alzheimer’s Association, 2021). However, despite these concerning statistics, there are currently no FDA-approved therapies that can prevent, reverse, nor delay AD progression and eventual death (Breijyeh and Karaman, 2020; Livingston et al., 2020; Yiannopoulou and Papageorgiou, 2020).

AD neuropathogenesis is complex and involves several major hallmarks including extracellular amyloid plaques (Hampel et al., 2021), intracellular neurofibrillary tangles (NFTs; DeTure and Dickson, 2019), synaptic loss (Griffiths and Grant, 2022), glial cell dysfunction (Hickman et al., 2008), and lipid droplet (LD) accumulation in microglia (Farmer et al., 2020). However, many other pathologies are associated with AD onset and progression, including decreased glucose metabolism, oxidative stress, neuroinflammation, altered lipid and metabolite composition of the brain, and age- and disease-associated changes in peripheral metabolism (Xu et al., 2016; Gibson et al., 2020). Until recently, the majority of FDA-approved AD drugs on the market include cholinesterase inhibitors and a single NMDA antagonist, which solely tackle the symptoms of AD and neglect the underlying mechanisms of the disease (Wang and Reddy, 2017; Breijyeh and Karaman, 2020; Kuns et al., 2022). Furthermore, these drugs do not cure nor reverse the disease, which leaves patients and their families feeling increasingly despondent (Wang and Reddy, 2017; Breijyeh and Karaman, 2020; Kuns et al., 2022).

So far, two anti-amyloid monoclonal antibodies (mAbs), Aducanumab and Lecanemab, have received approval from the FDA to treat early AD. Aducanumab is fast-track approved and lecanemab is traditionally approved (Food and Drug Administration, 2023; Reardon, 2023b). A third anti-amyloid mAb, Donanemab, is expected to be FDA-approved by the end of 2023 (Mislan, 2023). Anti-amyloid mAbs have shown the greatest improvements in AD outcomes to date, reduce plaque burden, and are the first disease-modifying treatments shown to slow disease progression and cognitive decline (Gregory, 2023). However, their overall effects on cognition are modest, their therapeutic windows may be narrow, and the antibodies are associated with potentially fatal side effects known as amyloid-related imaging abnormalities (ARIA; Shi et al., 2022). Fortunately, numerous emerging therapeutics for the treatment of AD are currently in clinical trials and provide promising alternative treatment strategies for patients living with this devastating disease. In recent years, glial cell dysfunction and lipid homeostasis have received increasing recognition as key players in AD pathophysiology. For example, strategies that augment the function of microglia (the key immune effector cells in the brain), such as Sargramostim/Leukine (Potter et al., 2021) and TREM2-targeting mAbs such as AL002 and ATV:TREM2 (Paul et al., 2021; Ward et al., 2021; van Lengerich et al., 2023), have the potential to modify many of the mechanisms underlying AD. In addition, interventions that remedy disordered glial lipid metabolism in the brains of AD patients such as the fatty acid synthase inhibitor CMS121 (Ates et al., 2020) also provide promise as emerging therapeutics with the capacity to resolve multiple facets of AD neuropathogenesis. Overall, the purpose of this review is to critically examine the currently available drugs and therapeutics and to evaluate the next generation of AD therapeutics in development.

## 2. AD stages, pathophysiology, and subtypes

There are four clinical AD stages (De-Paula et al., 2012; Breijyeh and Karaman, 2020). The first stage is the “pre-symptomatic”/“preclinical” or “mild cognitive impairment (MCI)” stage during which an individual may demonstrate moderate memory loss and early changes in the cerebral cortex and hippocampus but does not exhibit any issues carrying out their daily functions (Tabert et al., 2006; Dubois et al., 2016; Breijyeh and Karaman, 2020). Notably, not all individuals with MCI develop dementia (Tabert et al., 2006). During this stage, amyloid plaques start building up gradually in the cerebral cortex and NFTs accumulate in the anterior medial temporal lobe (Koychev et al., 2020). The second stage is “mild”/“early” AD during which patients begin demonstrating concentration issues, memory loss, spatial and temporal confusion, mood changes, and depression (Kumar and Tsao, 2019; Breijyeh and Karaman, 2020). Exacerbated cognitive decline at this stage is coupled with increased amyloid infiltration into multiple brain regions including the medial temporal lobe, entorhinal cortex, hippocampus, cingulate gyrus, amygdala, caudate, putamen, claustrum, thalamus, hypothalamus, and white matter, and NFTs spread to the amygdala, hippocampus, and thalamus (Koychev et al., 2020). The third stage is “moderate” AD where the disease infiltrates greater cerebral cortex territory, resulting in more intense memory loss, inability to recognize family and friends, loss of impulse control, and loss of the ability to read, write, and speak correctly (Kumar and Tsao, 2019; Breijyeh and Karaman, 2020). In this stage, amyloid plaques infiltrate the midbrain and medulla and NFTs are found in neocortical association cortices (Koychev et al., 2020). The fourth and final stage is “severe”/“late” stage AD during which the disease takes over the entirety of the cerebral cortex and amyloid plaques and NFTs dramatically increase in number (De-Paula et al., 2012; Apostolova, 2016; Breijyeh and Karaman, 2020). Late-stage patients fail to identify their family members, can become bedridden, and experience problems with swallowing and urinating, with complications ultimately leading to the patient’s death (De-Paula et al., 2012; Apostolova, 2016; Breijyeh and Karaman, 2020). It should be noted that pathophysiological changes in the brain occur decades before the onset of AD symptoms, which makes diagnosis and successful treatment of the disease even more challenging (Hof et al., 1996; Perl, 2010; Bateman et al., 2013; Beason-Held et al., 2013).

In terms of AD subtypes, two exist: early-onset AD (EOAD) and late-onset AD (LOAD; Bekris et al., 2010; Reitz and Mayeux, 2014). 5 to 10% of all AD cases are EOAD cases with individuals ranging from 30 to 65 years of age (Bekris et al., 2010; Reitz et al., 2020). The majority of EOAD and LOAD cases are sporadic (Reitz et al., 2020). LOAD is the most common subtype of AD and occurs in individuals 65 years of age and older, with more than 90% of AD patients having sporadic LOAD (Bekris et al., 2010). Around 60% of EOAD cases have multiple individuals in their family who suffer from AD and 13% of these cases are inherited in an autosomal dominant pattern with at least three generations affected (Campion et al., 1999; Brickell et al., 2006; Bekris et al., 2010). EOAD can also occur in families with LOAD (Bird, 2008; Bekris et al., 2010). Although there are a few families with monogenic AD, the vast majority of AD cases are the result of the interaction between multiple genes and environmental risk factors (Kamboh, 2004; Bird, 2008; Bekris et al., 2010).

## 3. AD genetic risk factors

Genome-wide association studies (GWAS) have identified 101 single nucleotide polymorphisms (SNPs) across 81 loci to be associated with sporadic LOAD (Andrews et al., 2023). Here, we discuss some of the more extensively studied variants. The following best characterized gene variants are associated with increased amyloid load (Raux, 2005; Bekris et al., 2010; Xiao et al., 2021). Variants in the *Presenilin-1* (*PSEN1*) gene*, Amyloid Precursor Protein* (*APP*) gene, and *Presenilin-2* (*PSEN2*) gene cause early-onset familial AD (EOFAD), with more than 200 mutations described worldwide (VIB-UAntwerp, 2018; Mendez, 2019; Qin et al., 2020; Xiao et al., 2021). *PSEN1* encodes Presenilin-1, the proteolytic subunit of γ-secretase, which is known to process APP (medlineplus.gov, 2021). *PSEN1* variants are the most “frequent cause” of AD (medlineplus.gov, 2021; Xiao et al., 2021) and increase secretase activity, leading to increased production of amyloid beta 42 (Aβ42; Wolfe, 2007). *PSEN1* mutations cause the most severe forms of AD and carriers can develop the disease as early as 30 years old (Bekris et al., 2010). In addition, *APP* variants represent 10 to 15% of EOFAD cases (Bird, 2008; Bekris et al., 2010). Most *APP* mutations are proximal to the γ-secretase cleavage site and are associated with elevated Aβ42 levels compared to other Aβ isoforms (Scheuner et al., 1996; Esler, 2001; Bekris et al., 2010). *PSEN2* variants may also increase γ-secretase activity and Aβ42 production but are less common than *PSEN1* variants and are associated with less aggressive disease progression (An et al., 2015; Xiao et al., 2021). Although the amyloid hypothesis of AD remains controversial, variants leading to altered APP processing have devastating effects on carriers and their families, thereby warranting further research in this area and personalized strategies that target aberrant APP processing (Kametani and Hasegawa, 2018). Additionally, variants associated with other facets of AD, namely lipid and lipoprotein processing, are becoming increasingly implicated in AD onset and severity.

The *Apolipoprotein E* (*APOE*) gene is the only gene that has been consistently associated with familial and sporadic LOAD, and three *APOE* alleles exist: ε2, ε3, and ε4 (Bekris et al., 2010). The ε4 allele confers high AD risk, an earlier age of onset for AD in Down syndrome, poorer outcomes following head trauma and stroke, and an increased risk of cardiometabolic disease (Nicoll et al., 1995; Liu et al., 2002; Bekris et al., 2010; Liu et al., 2019b). The least common ε2 allele of *APOE* confers a protective effect against AD whereas the most common ε3 allele appears to be neutral (Liu et al., 2013; Mayo Clinic Staff, 2021). ApoE is the major scaffold protein in brain-derived lipoproteins and plays a critical role in lipid and cholesterol transport (Mahley, 2016). *APOE* ε4 carriers have higher total cholesterol and LDL (low-density lipoprotein) levels (Sing and Davignon, 1985), greater amyloid and NFT pathologies (Huang, 2006), and greater mitochondrial damage compared to individuals with other *APOE* polymorphisms (Nagy et al., 1995; Gibson et al., 2000; Bekris et al., 2010). Around 25% of Caucasians carry at least one ε4 allele, with a 3-fold increased AD risk for heterozygotes and a nearly 15-fold increased AD risk for homozygotes (Gharbi-Meliani et al., 2021). However, up to 50% of individuals carrying at least one ε4 allele do not develop AD, which suggests that there are other genetic and environmental factors at play (Bekris et al., 2010).

Many other gene variants associated with AD encode proteins that regulate metabolism. For example, the *Sortilin-Related Receptor 1* (*SORL1*) gene is strongly associated with LOAD (Felsky et al., 2014). SORL1 regulates intracellular processing of factors involved in lipid metabolism and insulin signaling like lipoprotein lipase (LPL) and ApoE and regulates the endocytosis of LDL (Wong et al., 2010; Zollo et al., 2017; Su et al., 2021). SORL1 also transports the ATP-binding cassette transporter A1 (ABCA1), a lipid efflux transporter, into the lysosome for degradation, which causes a marked decrease in lipid efflux from macrophages and microglia (Willnow et al., 2011; Barthwal et al., 2013; Knupp et al., 2020; Su et al., 2021). Notably, rare heterozygous premature stop codon mutations in the *ABCA7* (ATP-binding cassette subfamily A member 7) gene, which encodes ABCA7 (another protein critical for lipid export), are associated with EOAD and LOAD risk (Bossaerts et al., 2022). ABCA7 is expressed in neurons, astrocytes, microglia, blood–brain barrier endothelial cells, and brain pericytes and plays a role in phagocytosis as well as the export of phospholipids in the presence of ApoA-1 and ApoE (Jehle et al., 2006; Tomioka et al., 2017; Dib et al., 2021; Bossaerts et al., 2022). Moreover, the C allele at the rs11136000 locus of the ApoJ or clusterin gene (*CLU* gene) is the strongest genetic risk factor for LOAD after the *APOE* ε4 allele and the rare R47H *TREM2* variant (Rajagopalan et al., 2013; Hooli et al., 2014; Roussotte et al., 2014). Importantly, Clusterin/ApoJ is a key component of brain-derived lipoproteins and is therefore critical to brain lipid transport and homeostasis (Merrill et al., 2023), and also plays important roles in immune response modulation, cell death and survival, oxidative stress, and proteotoxic stress (e.g., Aβ build-up and toxicity; Foster et al., 2019; Kim et al., 2022).

TREM2 (Triggering Receptor Expressed on Myeloid Cells 2) is a transmembrane receptor that is activated by a variety of ligands including lipids (Cannon et al., 2011; Gratuze et al., 2018) and lipoproteins (Wang et al., 2015; Yeh et al., 2016; Gratuze et al., 2018). TREM2 is essential for microglial clearance of damaged myelin and a loss of TREM2 leads to the downregulation of lipid metabolism genes (Cantoni et al., 2015; Keren-Shaul et al., 2017; Griciuc et al., 2019; Schlepckow et al., 2020). Loss-of-function *TREM2* variants are associated with increased AD risk (van Lengerich et al., 2023) and augment the risk of developing LOAD by 2 to 4 times – similar to the risk associated with *APOE* ε4 heterozygosity (Corder et al., 1993; Gratuze et al., 2018). Furthermore, TREM2 expression is upregulated in aging and AD (Lue et al., 2015; Perez et al., 2017; Gratuze et al., 2018). TREM2 also binds C1q with high affinity, which leads to a dampening of the complement cascade’s activation (Zhong et al., 2023). Since, in the brain, TREM2 is exclusively expressed in microglia (the brain’s key immune effector cells), and its activation can regulate the immunometabolic polarization of these cells, it makes sense that TREM2 plays an important role in the immunomodulatory mechanisms driving AD (Li and Zhang, 2018). Altogether, the robust association between ApoE, SORL1, and TREM2 highlights the important role of microglial-lipid and lipoprotein processing in the neuropathogenesis of AD.

In further support of the notion that lipid processing in the brain is critical to AD, a recent study reported a novel protective variant called the *RELN-COLBOS* variant (Lopera et al., 2023). In adults, RELN (Reelin) is expressed in GABAergic interneurons of the forebrain, glutamatergic neurons of the entorhinal cortex, olfactory mitral cells, and granule cells of the cerebellum (Doehner and Knuesel, 2010). *RELN* encodes Reelin, a glycoprotein that plays an important role in synaptic function and is a ligand for lipoprotein receptors such as very low-density lipoprotein receptor (VLDLR) and apolipoprotein E receptor 2 (ApoER2; D’Arcangelo et al., 1999; Ishii et al., 2016; Lopera et al., 2023). Whether reelin is a component of brain-derived lipoproteins or other lipid-containing extracellular vesicles is unclear. The *RELN-COLBOS* variant was identified by researchers investigating individuals that carry the *PSEN1-E280A* variant, which usually leads to MCI by age 44 (median age) and dementia by age 49, with exceptions being very rare (Lopera et al., 2023). Lopera et al. presented the case of a male who was protected against the *PSEN1-E280A* variant by carrying two copies of *RELN-COLBOS*. The male was cognitively normal until 67 and was diagnosed with MCI at 70, but eventually died of dementia-associated aspiration pneumonia at the age of 74. Analysis of his brain revealed increased glucose metabolism in the precuneus and whole brain, high amyloid plaque burden in the cortex, and significantly low tau levels in the entorhinal cortex, posterior cingulate cortex, and precuneus. Lopera et al. suggest that low tau levels in the entorhinal cortex are responsible for the *RELN-COLBOS* variant’s protective effect. However, further studies may be needed to determine the exact mechanism and causality. This was the second “ascertained extreme resilience to autosomal dominant Alzheimer’s disease (ADAD)” case in the world (Lopera et al., 2023). The first case, also discovered by the same research group, was a female who carried the pathological *PSEN1-E280A* mutation as well as two copies of the protective *APOE3* Christchurch (*APOECh*; R136S) variant (Arboleda-Velasquez et al., 2019). This woman did not experience cognitive issues until around 30 years after the expected clinical onset age (Arboleda-Velasquez et al., 2019). *APOECh* decreases ApoE-LDL receptor binding, which likely alters lipid and cholesterol transport (Lalazar et al., 1988; Mahley et al., 1999; Arboleda-Velasquez et al., 2019). It is important to note that the aforementioned mutations are not the only protective mutations associated with AD. Therapeutics that mimic the effects of protective mutations by imitating their mechanisms of action, potentially by restoring lipid and lipoprotein homeostasis in the AD brain, are an exciting and promising future research direction for AD treatment.

## 4. Current FDA-approved AD neurotransmitter-targeting drugs: a symptomatic fix

There are currently two neurotransmitter-targeting drug classes approved to treat AD: cholinesterase inhibitors and a single NMDA (N-methyl-D-aspartate) antagonist (Breijyeh and Karaman, 2020). Although these drugs slightly improve cognition, their overall effects are modest (Xu et al., 2021). Furthermore, there is no clinical evidence to show that any of these drugs provides benefits to patients beyond 1 year, with some patients not benefiting at all (Sackett, 2018). Also, these drugs do not delay nor prevent patients from becoming institutionalized nor do they enhance the patient’s quality of life (Sackett, 2018).

Cholinergic transmission is reduced in AD due to the degeneration of cholinergic neurons (Fahnestock and Shekari, 2019). Inhibiting the cholinesterase enzymes acetylcholinesterase and butyrylcholinesterase increases acetylcholine levels in the synaptic cleft, which is thought to mitigate the effects of cholinergic neuron degeneration (Colovic et al., 2013). Current FDA-approved cholinesterase inhibitors include donepezil, rivastigmine, and galantamine (Anand and Singh, 2013; Breijyeh and Karaman, 2020). Donepezil is an orally administered reversible inhibitor of acetylcholinesterase and is well-tolerated with moderate, fleeting side effects such as diarrhea, loss of appetite, muscle cramps, nausea, sleeping problems, tiredness or weakness, and vomiting (Knowles, 2006; IBM Micromedex, 2022). Donepezil improves cognition and behavior in the “short to medium term” but does not affect the progression of AD (Knowles, 2006). It is prescribed for patients with mild-to-moderate or severe AD (Birks and Harvey, 2018).

Rivastigmine is a pseudo-irreversible inhibitor of acetylcholinesterase and butyrylcholinesterase (Birks et al., 2015). It is “pseudo-irreversible” because it forms a covalent bond with the enzymes’ catalytic site and the bond is eventually spontaneously hydrolyzed (Groner et al., 2007; Moss et al., 2016). Rivastigmine is reserved for mild-to-moderate AD cases and only slightly enhances cognitive function and daily activity performance (Birks et al., 2015). Oral administration of the drug can cause side effects including nausea, vomiting, indigestion, tiredness or weakness, anorexia, and weight loss (Inglis, 2002; Breijyeh and Karaman, 2020). Rivastigmine can also be administered transdermally using a patch, which decreases side effects (Birks et al., 2015).

Galantamine is a reversible acetylcholinesterase inhibitor that also allosterically modulates nicotinic cholinergic receptors and increases acetylcholine’s effects at these receptors to further augment cholinergic transmission (Harvey, 1995; Maelicke et al., 1997). Galantamine improves behavior, daily activity performance, and cognition (Loy and Schneider, 2006). It is well-tolerated and is administered orally via tablet or liquid (Loy and Schneider, 2006; WebMD, 2019). Side effects include nausea, vomiting, diarrhea, dizziness, drowsiness, loss of appetite, and weight loss (WebMD, 2019). Galantamine is the only cholinesterase inhibitor shown to significantly decrease patients’ risk of developing severe dementia (Xu et al., 2021). Galantamine is used in mild-to-moderate AD patients (Loy and Schneider, 2006).

NMDA glutamate receptors play an important role in AD (Liu et al., 2019a). NMDA receptors become overactive, which leads to abnormal synaptic calcium “handling” (Mota et al., 2014; Liu et al., 2019a) and excitotoxicity (Rothman and Olney, 1986; Choi, 1987; Sattler et al., 2000). NMDA antagonists inhibit NMDA glutamate receptor overactivation and excitotoxicity, thereby preserving neuronal function and cognition (Breijyeh and Karaman, 2020). Memantine is the only available NMDA antagonist for AD treatment (Parsons et al., 2013). It is used for the treatment of moderate-to-severe AD (Parsons et al., 2013). It is an uncompetitive NMDA receptor antagonist that binds with moderate affinity (Parsons et al., 1993; Danysz et al., 2000). The drug is well-tolerated with side effects including dizziness, headache, diarrhea, constipation, and confusion (Parsons et al., 1999; National Institute on Aging, 2021). It can be administered orally via tablet or liquid (National Institute on Aging, 2021). Similar to cholinesterase inhibitors, memantine does not slow nor reverse the neuropathogenesis of AD. Memantine can be used alone or in combination with donepezil (Farrimond et al., 2012). It is recommended that donepezil and memantine be taken as a combination therapy since they work in a synergistic fashion (Parsons et al., 2013). This is supported by the fact that the glutamatergic and cholinergic systems influence each other (Parsons et al., 2013).

Overall, cholinesterase inhibitors and memantine stabilize cognition for 2 to 5 months before the patient’s cognition declines again (Vaci et al., 2020). Since these neurotransmitter-modulating drugs fail to provide patients with any benefits beyond a few months or a single year at best, pharmaceutical companies have developed anti-amyloid mAbs in the hopes that a decreased amyloid plaque burden would correlate with improved cognition in AD patients.

## 5. The latest anti-amyloid mAbs: Aducanumab, Lecanemab, and Donanemab

**Figure 1:**
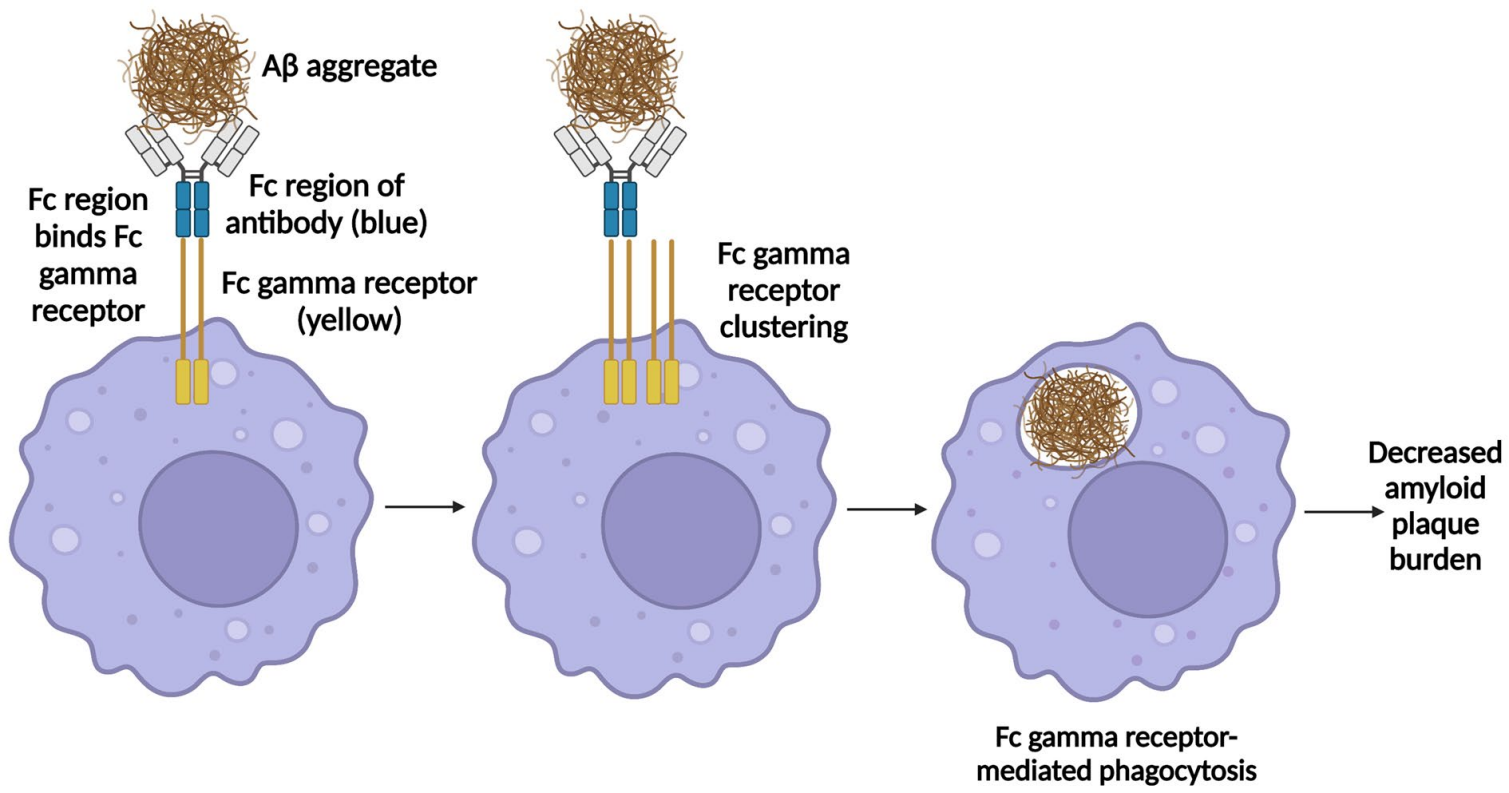
Mechanism of action of Aducanumab, Lecanemab, and Donanemab. Aducanumab binds oligomeric and fibrillary amyloid aggregates, lecanemab binds soluble Aβ protofibrils, and donanemab binds N-terminally truncated pyroglutamate-modified Aβ (Aβ_pE_), a type of Aβ only found in plaques in the brain and not in cerebrospinal fluid (CSF) nor plasma. Once each antibody binds its respective target, the Fc region of the antibody binds Fc gamma receptors (FCGRs) on phagocytes such as microglia and perivascular macrophages, which induces FCGR clustering, leading to FCGR-dependent phagocytosis, thereby enabling antibody clearance of Aβ from the brain. Information derived from Garapati (2012), Alawode et al. (2021), Lowe et al. (2021), Haddad et al. (2022), Söderberg et al. (2022), and van Dyck et al. (2023). Created with BioRender.com.

Recently, three anti-amyloid antibodies, Aducanumab, Lecanemab, and Donanemab, have been developed (Garapati, 2012; Lowe et al., 2021; Söderberg et al., 2022). While there are subtleties in their mechanisms of action, for each mAb the Fc region of the antibody binds Fc gamma receptors (FCGRs) on phagocytes such as microglia and perivascular macrophages, which causes the FCGRs to cluster, leading to FCGR-dependent phagocytosis (Garapati, 2012) and clearance of Aβ from the brain (Figure 1).

Biogen’s Aducanumab is the first anti-amyloid mAb to be fast-track approved by the FDA (Mullard, 2023). It selectively targets oligomeric and fibrillary Aβ aggregates and reduces plaque burden in AD patient brains, which is thought to slow cognitive decline (Sevigny et al., 2016; Budd Haeberlein et al., 2022; Haddad et al., 2022). Two phase 3 clinical trials, EMERGE (ClinicalTrials.gov, 2015b) and ENGAGE (ClinicalTrials.gov, 2015a), were conducted in subjects with MCI and early AD (Budd Haeberlein et al., 2022). Subjects were randomized to 3 or 6 mg/kg aducanumab (low dose), 10 mg/kg aducanumab (high dose), or placebo, all of which were administered via IV infusion once every 4 weeks over the course of 76 weeks (Budd Haeberlein et al., 2022). The primary outcome of both studies was the change from baseline to week 78 in Clinical Dementia Rating Sum of Boxes (CDR-SB) scores. Both trials were terminated due to “futile” data combined from 50% of subjects first enrolled, and analyses of efficacy were based on a larger data set gathered up to “futility declaration.” The EMERGE study showed statistically significant changes in CDR-SB scores (*p*-value: 0.012; 22% decrease in score), Mini-Mental State Examination (MMSE) scores (*p*-value: 0.049; 18% decrease in score), Alzheimer’s Disease Assessment Scale-Cognitive Subscale version 13 (ADAS-Cog 13) scores (*p*-value: 0.010; 27% decrease in score), and Alzheimer’s Disease Cooperative Study-Activities of Daily Living Scale for use in Mild Cognitive Impairment (ADCS-ADL-MCI) scores (*p*-value < 0.001; 40% decrease in score) for the high aducanumab dose only. The ENGAGE study failed to meet these same endpoints for both low and high aducanumab doses. Furthermore, 26% of subjects in the low dose group and 35% of subjects in the high dose group had amyloid-related imaging abnormalities due to edema (ARIA-E) in the EMERGE study, and 26% of subjects in the low dose group and 36% of subjects in the high dose group had ARIA-E in the ENGAGE study. Amyloid-related imaging abnormalities due to hemorrhage (ARIA-H) occurred in 19% of subjects in the high dose group of the EMERGE study and in 6% of subjects in the high dose group of the ENGAGE study (Budd Haeberlein et al., 2022). In addition, four deaths have been linked to aducanumab so far (Dunleavy, 2022). Aducanumab is currently in a phase 3b/4 clinical trial (ENVISION) to “verify the clinical benefit” of the antibody in early AD patients, with the trial set to end on October 31, 2026 (ClinicalTrials.gov, 2022c).

Biogen’s Lecanemab is the second anti-amyloid antibody to be fast-track approved and the first to receive full approval by the FDA (Food and Drug Administration, 2023; Mullard, 2023). It binds soluble Aβ protofibrils, which are thought to be the most neurotoxic form of Aβ, and stops their deposition and the subsequent formation of amyloid plaques (Haass and Selkoe, 2007; Lublin and Gandy, 2010; Tucker et al., 2015; Hampel et al., 2021; van Dyck et al., 2023). An 18-month phase 3 clinical trial called Clarity AD (ClinicalTrials.gov, 2019) was conducted in subjects with MCI and early AD (van Dyck et al., 2023). Subjects received either 10 mg/kg of lecanemab every 2 weeks via IV or undisclosed placebo (van Dyck et al., 2023). The primary endpoint was the change in CDR-SB scores from baseline at 18 months and secondary endpoints were the change from baseline at 18 months in PET (positron emission tomography) scan amyloid burden, ADAS-Cog14 scores, Alzheimer’s Disease Composite Scores (ADCOMS), and ADCS-MCI-ADL scores. Lecanemab caused an overall decrease of 0.45 points in CDR-SB scores (*p*-value < 0.001), an overall decrease of 1.44 points in ADAS-Cog14 scores (*p*-value < 0.001), an overall decrease of 0.050 points in ADCOMS (*p*-value < 0.001), and an overall increase of 2 points in ADCS-MCI-ADL scores (*p*-value < 0.001). Lecanemab also caused an overall decrease of 59.12 centiloids in amyloid burden (*p*-value < 0.001). However, ARIA-E occurred in 12.6% of lecanemab subjects compared to 1.7% of placebo subjects, and ARIA-H occurred in 17.3% of lecanemab subjects compared to 9.0% of placebo subjects. 8.2% of lecanemab subjects experienced ARIA-E and ARIA-H at the same time compared to 1.0% of placebo subjects (van Dyck et al., 2023). Furthermore, lecanemab has caused three patient deaths so far in the extended phase of the Clarity AD phase 3 trial due to ARIA-H and seizures (Reardon, 2023b). Lecanemab weakened their cerebral blood vessels upon attacking amyloid plaques lining the blood vessels. All three patients were taking anticoagulants, which further exacerbated their brain bleeds (Reardon, 2023b). The extension phase of the Clarity AD trial is currently ongoing and is set to end on September 15, 2027 (ClinicalTrials.gov, 2019).

Eli Lilly’s Donanemab is the third latest anti-amyloid antibody, and it works by targeting N-terminally truncated pyroglutamate-modified Aβ (Aβ_pE_), a type of Aβ only found in plaques in the brain and not in cerebrospinal fluid (CSF) nor plasma (Harigaya et al., 2000; DeMattos et al., 2012; Alawode et al., 2021; Mintun et al., 2021; Reardon, 2023a). Donanemab is expected to be FDA-approved by the end of 2023 (Mislan, 2023). In the TRAILBLAZER-ALZ 2 phase 3 trial (ClinicalTrials.gov, 2020b), donanemab subjects received 700 mg of donanemab every 4 weeks for three doses followed by 1,400 mg of donanemab via IV every 4 weeks with a “potential blinded dose reduction to placebo” depending on amyloid plaque clearance at 24 and 52 weeks (Zimmer et al., 2022). Subjects with intermediate and high levels of tau were recruited and subjects were stratified based on cerebral tau levels (Zimmer et al., 2022; Eli Lilly and Company, 2023). The primary endpoint of the trial was the change from baseline to 76 weeks in Integrated Alzheimer’s Disease Rating Scale (iADRS) scores and secondary endpoints included changes in CDR-SB scores and other cognitive assessments, amyloid PET scans, tau PET scans, and volumetric MRI (magnetic resonance imaging) scans (Zimmer et al., 2022; Eli Lilly and Company, 2023). iADRS scores showed that donanemab slowed cognitive decline by 35% from baseline until 18 months (*p*-value < 0.0001) and CDR-SB scores demonstrated that donanemab slowed cognitive decline by 36% over 18 months (*p*-value < 0.0001; Eli Lilly and Company, 2023). Alzheimer’s Disease Cooperative Study – Instrumental Activities of Daily Living Inventory (ADCS-iADL) scores revealed that donanemab subjects experienced a 40% less decline in daily living activity performance at 18 months (*p*-value < 0.0001), and CDR global scores determined that donanemab subjects had a 39% lower risk of progressing to the subsequent AD stage compared to placebo subjects (*p*-value < 0.001). 52% of subjects completed donanemab treatment by 1 year and 72% completed treatment by 18 months once a prespecified and undisclosed amyloid clearance threshold was met. Similar to aducanumab and lecanemab, ARIA was increased in donanemab treatment groups – specifically, 24% of donanemab subjects experienced ARIA-E and 31.4% experienced ARIA-H while 13.6% of placebo subjects experienced ARIA-H. Donanemab has caused three deaths in the TRAILBLAZER-ALZ 2 trial as a result of ARIA (Eli Lilly and Company, 2023) and is currently in three other clinical trials: TRAILBLAZER-ALZ 3 (ClinicalTrials.gov, 2021a), TRAILBLAZER-ALZ 5 (ClinicalTrials.gov, 2022a), and TRAILBLAZER-ALZ 6 (ClinicalTrials.gov, 2023).

Since ARIA is the most common adverse side effect of the anti-amyloid immunotherapies, it is important to consider the mechanisms leading to ARIA onset when evaluating the efficacy of these treatments as well as the candidacy of those receiving treatments. ARIA-E consists of vasogenic edema and extravasated fluid and ARIA-H consists of microhemorrhages and hemosiderosis (Sperling et al., 2011). Extravasated fluid refers to protein-containing fluid that leaks from meningeal vessels into the leptomeningeal or sulcal space. Vasogenic edema tends to occur in the gray and white matter and is caused by higher permeability of brain capillary endothelial cells to serum proteins, resulting in a greater volume of extracellular fluid. As for ARIA-H, microhemorrhages are small iron deposits in tissue in the form of hemosiderin which occur as lesions and small volumes of blood in the brain parenchyma (Linn et al., 2010; Sperling et al., 2011). ARIA-E and ARIA-H are thought to occur when anti-amyloid mAbs bind to Aβ deposits in cerebral parenchyma and vessels (Roytman et al., 2022), thereby weakening vessel walls and causing vessels to leak protein-containing fluids (ARIA-E) and heme products (ARIA-H; Sperling et al., 2011; Barakos et al., 2022; Roytman et al., 2022). Although the exact pathological mechanisms remain an area of active investigation, several *ex vivo* studies have suggested that anti-amyloid mAbs lead to microglial activation (Ostrowitzki et al., 2011; Bohrmann et al., 2012; Sevigny et al., 2016). Microglial recruitment and perivascular macrophages presumably result in Aβ clearance as well as the walling off of plaques to protect nearby cells. However, it is plausible that microglial recruitment could initiate a maladaptive inflammatory cascade that leads to a breakdown of the blood–brain barrier (BBB) and weakened vessels, which contribute to ARIA (Sperling et al., 2011; Chen et al., 2017; Roytman et al., 2022). In support of this, a recent *in vivo* study used positron emission tomography (PET) and _11_C-PK11195, a tracer targeting the 18-kDa translocator-specific protein overexpressed in activated microglia cells, and showed that activated microglia are associated with ARIA-E (Cagnin et al., 2001; Papadopoulos et al., 2006; Cerami et al., 2017; Piazza et al., 2022). Further studies that define the neurometabolic mechanisms of ARIA and resolve what constitutes “active” and “maladaptive” microglia will be critical in predicting which patients are more at risk of adverse side effects.

Overall, these anti-amyloid mAbs reduce amyloid burden and slow cognitive decline and AD progression for around 18 months, which has a huge impact on the quality of life for patients, families, and caregivers. Lecanemab and donanemab are, therefore, major breakthroughs in AD treatment and set the stage for a new standard of care that incorporates disease-modifying strategies. However, neurologists are skeptical about the impact of these antibodies on the AD patient’s day-to-day life (Reardon, 2023a). The improvements in the cognitive scale measures are predicted to be almost unnoticeable in a patient’s daily life, which poses the following question: Is it worth risking potentially fatal ARIA occurrences for a marginal therapeutic benefit (Reardon, 2023a)? In addition, anti-amyloid mAbs are expensive and may not be available to all patients. Currently, aducanumab is priced at $28,200 annually (Hoffman, 2021), lecanemab is priced at $26,500 annually (Cubanski and Neuman, 2023), and donanemab is expected to be priced between $26,500 and $39,000 annually (Macfarlane, 2023). Nonetheless, since amyloid burden may be a critical component of disease progression (particularly in individuals with EOFAD and Down syndrome), non-antibody anti-amyloid therapeutics that do not cause ARIA are of great interest.

## 6. CT1812: selective sigma-2 receptor antagonist that displaces Aβ oligomers from synaptic receptors

**Figure 2:**
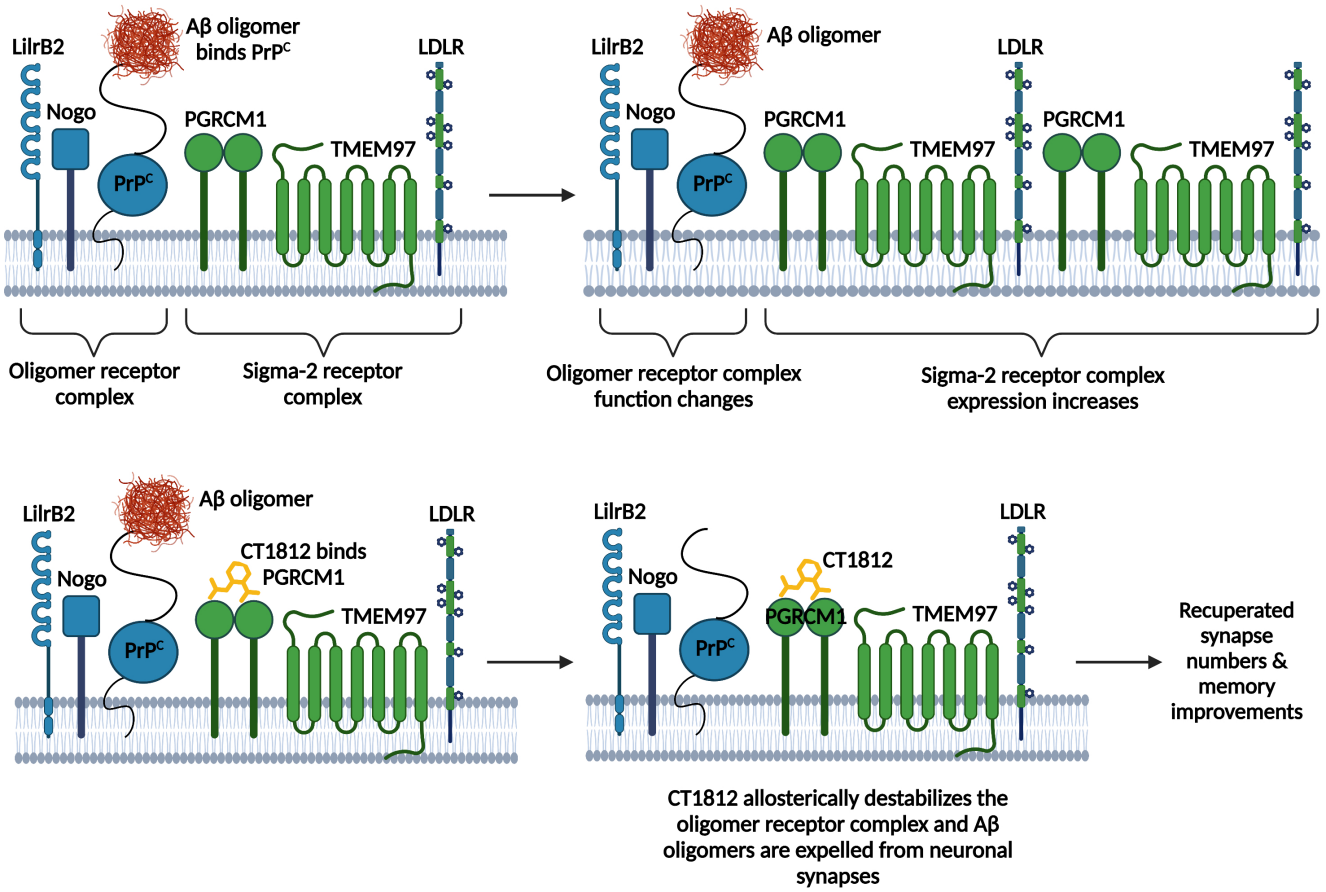
CT1812’s mechanism of action. The Aβ oligomer receptor complex consists of LilrB2 (leukocyte immunoglobulin-like receptor B2), the Nogo receptor, and PrP^C^ (cellular prion protein). The sigma-2 receptor complex consists of PGRMC1 (Progesterone Membrane Binding Component-1), the ligand binding site of the complex, TMEM97 (Transmembrane Protein 97), and the LDLR (Low-Density Lipoprotein Receptor). Aβ oligomers bind PrP^C^ on the oligomer receptor complex, which changes the function of the complex and increases the expression of the sigma-2 receptor complex. CT1812 binds PGRMC1, which allosterically regulates the sigma-2 receptor complex and destabilizes adjacent Aβ oligomer receptor binding sites, which stops Aβ oligomers from binding their receptors and allows for their eventual clearance into CSF. Figure adapted from Izzo et al. (2021). Information derived from Xu et al. (2011), Izzo et al. (2014a,b, 2021), Jarosz-Griffiths et al. (2016), Mroczko et al. (2018), and Riad et al. (2020). Created with BioRender.com.

Aβ oligomers are one of the most toxic structural forms of Aβ and cause synaptotoxicity and memory decline as they accumulate in the brain (Tomic et al., 2009; Bao et al., 2012; Esparza et al., 2012; Izzo et al., 2021). Sigma-2 receptor (σ2R)/TMEM97 (Transmembrane Protein 97) antagonists can reduce Aβ oligomer toxicity (Riad et al., 2018, 2020; Izzo et al., 2021). PGRMC1 (Progesterone Membrane Binding Component-1) is the ligand binding site of the σ2R/TMEM97 complex (Xu et al., 2011). TMEM97 and PGRMC1 form a trimeric complex with the LDLR (Low-Density Lipoprotein Receptor) to allow for the swift internalization of lipoproteins LDL and ApoE (Riad et al., 2018, 2020). The TMEM97/PGRMC1/LDLR complex is required for the uptake of monomeric and oligomeric Aβ42 and ApoE via both ApoE-dependent and -independent mechanisms (Riad et al., 2018, 2020). σ2R antagonists like CT1812 appear to be neuroprotective in AD (Yang et al., 2020; Izzo et al., 2021). CT1812 is the first selective σ2R antagonist to reach clinical trials and has proven to be successful in displacing Aβ oligomers from synaptic receptors (Grundman et al., 2019; Izzo et al., 2021). CT1812 binds PGRMC1 and allosterically regulates the σ2R complex and destabilizes adjacent Aβ oligomer receptor binding sites, which effectively stops Aβ oligomers from binding their receptors and thus allows for their eventual clearance into CSF (Izzo et al., 2014a,b, 2021; Mroczko et al., 2018; Riad et al., 2020; Figure 2). Interestingly, reduced binding of Aβ oligomers to synaptic receptors is one of the proposed mechanisms underlying the protective Icelandic mutation (Limegrover et al., 2020; Izzo et al., 2021). The Icelandic mutation (A673T mutation) is the only protective AD mutation that markedly decreases AD occurrence and significantly lowers the binding affinity of Aβ oligomers at synaptic receptors (Jonsson et al., 2012; Limegrover et al., 2020; Izzo et al., 2021). Aβ oligomer binding hindrance has been associated with better membrane trafficking, augmented synaptic protein expression, less spine loss, and ameliorated cognitive decline in AD animal models (Izzo et al., 2014a,b, 2021). Therefore, CT1812 likely mimics the protective effects of this variant.

*In vitro* studies using primary rat neuronal and glial cultures have shown that CT1812 can reverse the membrane trafficking deficits inflicted by synthetic Aβ oligomers (Izzo et al., 2021). The same effect was observed when Aβ oligomers derived from human AD patient brains were used (*p*-value < 0.001). CT1812 prevented Aβ oligomers from binding to synaptic receptor sites and displaced any Aβ oligomers that were already bound, which led to increased synaptic density and improved synaptic protein expression (neurogranin and synaptotagmin-1) in cultured mouse neurons. Supplementation of Aβ oligomers to cultured rat neurons resulted in a 28% loss of neurons expressing augmented levels of neurogranin (*p*-value: 0.014) and a 37% loss of synaptotagmin-1 presynaptic terminals (*p*-value: 0.0068). CT1812 treatment restored the expression of both proteins to control levels (Izzo et al., 2021). Furthermore, as the concentration of CT1812 increased, more Aβ oligomers were released from postmortem neocortical tissue from AD patients (*p*-value < 0.05; Izzo et al., 2021).

*In vivo*, CT1812 treatment resulted in a rapid and marked increase in Aβ oligomer levels in the interstitial fluid (ISF) and CSF of AD mice compared to pre-dose baseline, thereby confirming brain clearance (*p*-value < 0.0001; Izzo et al., 2021). CT1812 had no effect on Aβ monomer levels in the CSF, confirming that CT1812 only targets oligomers for brain clearance. In an aged transgenic AD mouse model, CT1812 treatment led to improvements in spatial learning (assessed using the Morris Water Maze and Y-Maze), memory (assessed using contextual fear conditioning), hyperactivity (assessed using the Activity Chamber assay), and cue-dependent learning (assessed using the Fear Conditioning assay). Healthy wild-type mice were unaffected by CT1812.

Considering successful *in vitro* and *in vivo* findings, CT1812 has been taken through to clinical studies. CT1812 was found to be safe and well-tolerated in a phase 1 clinical trial of 80 healthy, young and elderly individuals (ClinicalTrials.gov, 2015c; Grundman et al., 2019). In another phase 1b/2a trial (ClinicalTrials.gov, 2016), 19 mild-to-moderate AD patients were randomized to receive 1 of 3 CT1812 doses (90, 280, or 560 mg) or an unspecified placebo once a day for 28 days to determine CT1812’s safety and tolerability (Izzo et al., 2021). CT1812 was generally safe and well-tolerated in this trial, but four individuals experienced lymphocytopenia (Jeremic et al., 2021). At the end of treatment, Aβ oligomer CSF concentrations in placebo subjects were lower than baseline and Aβ oligomer CSF concentrations in CT1812 subjects were markedly increased compared to placebo subjects (*p*-value: 0.014; Izzo et al., 2021). Aβ40 and Aβ42 monomer levels did not differ between day 0 and day 28 nor between CT1812- and placebo-treated groups, as expected. At the end of treatment, CT1812 subjects had lower neurogranin (*p*-value: 0.050) and synaptotagmin-1 (*p*-value: 0.011) CSF levels compared to AD subjects treated with placebo (Izzo et al., 2021). This is a positive effect since neurogranin and synaptotagmin-1 in CSF are indicative of synaptic degeneration (Portelius et al., 2015; Öhrfelt et al., 2016). Synaptic proteome protein analysis of the CSF of the AD patients showed a significant increase in proteins associated with neurotransmission/signal transduction, thereby indicating CT1812’s favorable effect on synaptic activity (Izzo et al., 2021). CT1812 was also found to have a beneficial effect on proteins possibly involved in dendritic branching, cytoskeletal remodeling, and neurotransmission. AD subject CSF was also evaluated for unphosphorylated and phosphorylated tau protein fragments at baseline and end of treatment. At the end of treatment, the abundance of 6 tau phosphorylation sites dropped by 30% or more after CT1812 treatment in comparison with placebo, and the abundance of a single phosphorylation site rose by more than 30%. Unphosphorylated tau concentrations were unaffected. Phosphorylated tau and total tau changes from baseline were comparable between CT1812- and placebo-treated groups. Furthermore, the concentrations of kinases that phosphorylate tau protein like GSK-3β (glycogen synthase kinase-3 beta) dropped by 25% in CT1812 subjects, but this did not differ significantly from placebo subjects (*p*-value: 0.098; Izzo et al., 2021).

Cognition Therapeutics completed CT1812’s phase 2 clinical trial (ClinicalTrials.gov, 2021c), which assessed the effect of CT1812 on synaptic activity in 16 subjects with mild-to-moderate AD (results are currently unavailable), and the therapeutic is currently in another phase 2 clinical trial (ClinicalTrials.gov, 2022e) to assess CT1812’s safety, tolerability, and efficacy in 120 subjects with mild-to-moderate dementia with Lewy bodies, with the trial set to end on April 15, 2024. CT1812 boasts promising findings so far and is predicted to perform favorably in ongoing and future trials. However, therapeutics that only target Aβ may miss many of the other mechanisms underlying AD, such as neuroinflammation, glial cell dysfunction, and altered lipid and lipoprotein metabolism. Indeed, LDLRs are involved in AD pathogenesis and play a role in Aβ metabolism (Kim et al., 2009; Basak et al., 2012; Kanekiyo and Bu, 2014). Therapeutics targeting dyslipidemia and microglial activity via targeting key microglial lipoprotein receptors such as TREM2 are currently in clinical trials and are set to make a significant impact on the lives of AD patients.

## 7. ATV:4D9, ATV:TREM2, and AL002: agonistic TREM2 antibodies

TREM2 is predominantly expressed in microglia of the brain, where it modulates important microglial functions including phagocytosis (Kleinberger et al., 2014; Sierra et al., 2014; Keren-Shaul et al., 2017), migration (Mazaheri et al., 2017), lipid processing (Nugent et al., 2020), proliferation, lysosomal degradation, metabolism, and neuroinflammation (Keren-Shaul et al., 2017; van Lengerich et al., 2023). Notably, *TREM2* gene variants markedly increase the risk of developing LOAD (Ulland and Colonna, 2018). Aβ binds TREM2 to activate downstream signaling, which is more pronounced in response to oligomeric Aβ (Zhao et al., 2018). *In vitro* studies have shown that microglia lacking TREM2 demonstrate less phagocytosis of “apoptotic neurons, cellular debris, and bacteria or bacterial products,” and that increased TREM2 expression enhances the phagocytosis rate (Takahashi et al., 2005; Hsieh et al., 2009; N’Diaye et al., 2009; Kleinberger et al., 2014; Gratuze et al., 2018). *TREM2* mutations render microglia less able to surround amyloid fibrils and plaques, which leads to larger plaques, increased exposure to neighboring neurites, and neurotoxicity (Condello et al., 2015; Yuan et al., 2016). Hence, insufficient TREM2 levels hinder the “neuroprotective microglial barrier” that modulates the “compaction and insulation” of Aβ (Condello et al., 2015; Yuan et al., 2016). TREM2 also regulates the “switch” from homeostatic microglia to disease-associated microglia (DAM state microglia; Krasemann et al., 2017; Gratuze et al., 2018). TREM2 deficiencies also equate to reduced microglial proliferation and survival (Wang et al., 2016; Zheng et al., 2017; Gratuze et al., 2018).

Schlepckow et al. (2020) hypothesized that enhancing TREM2-mediated signaling would magnify the microglial functions that modify disease. To this end, they developed a way to propagate signaling by selectively inhibiting TREM2 cleavage. A disintegrin and metalloproteinase 10 and 17 (ADAM10 and ADAM17) are types of α-secretases involved in cleaving TREM2 (Asai et al., 2003; Kuhn et al., 2016; Schlepckow et al., 2020) within TREM2’s stalk region (Feuerbach et al., 2017; Schlepckow et al., 2017; Thornton et al., 2017). 4D9, a rat IgG2a antibody, binds near the ADAM10/17 cleavage site to prevent shedding and maintain TREM2 levels at the cell surface (Schlepckow et al., 2020). 4D9 also leads to TREM2 clustering on the surface of the cell, thereby triggering TREM2-mediated microglial activation via p-SYK signaling. 4D9’s effects were consistent in membrane fractions of bone marrow-derived macrophages (BMDMs), where it promoted the survival of BMDMs derived from heterozygous TREM2 knockout mice, suggesting that 4D9 promotes cell survival even if there is a partial loss of TREM2 function. Furthermore, 4D9 significantly increased microglial uptake of Aβ42 as well as myelin, suggesting that 4D9 can promote microglial phagocytosis of factors not limited to Aβ. 4D9 augmented TREM2 brain levels in a dose-dependent fashion in wild-type mice, which shifted microglial polarization from a homeostatic state to a DAM state and decreased total plaque levels in the cortices of *APP* mice (Schlepckow et al., 2020).

Van Lengerich et al. further developed 4D9 by adding a monovalent transferrin receptor (TfR) binding site called the antibody transport vehicle (ATV) in the Fc domain to give rise to ATV:4D9 with increased BBB (blood–brain barrier) penetrance (Kariolis et al., 2020; van Lengerich et al., 2023). ATV:4D9 caused higher microglial co-localization than 4D9, showing that ATV:4D9 has superior microglial targeting. ATV:4D9 treatment also caused microglia to shift to non-homeostatic DAM-like states and enhanced the diversity of microglial states in *App*^SAA^ knock-in TfR^mu/hu^ mice. Notably, ATV:4D9 also decreased soluble TREM2 levels in the CSF of wild-type;TfR^mu/hu^ mice (van Lengerich et al., 2023).

Following the promising results from preclinical AD models, the researchers then generated an antibody specific to human TREM2 with properties similar to those of ATV:4D9 (van Lengerich et al., 2023). The antibody was engineered with an effectorless Fc and ATV and was named ATV:TREM2. ATV:TREM2 binds the stalk region of TREM2 and induces TREM2 aggregation and endocytosis to strengthen TREM2 signaling. Specifically, the ATV converts a bivalent antibody into a tetravalent one and increases TREM2 signaling 100-fold (Burke, 2023). ATV also triggers formation of the TfR-TREM2 receptor complex, its internalization, and endosomal TREM2 signaling (van Lengerich et al., 2023). ATV:TREM2 enhances p-SYK signaling and promotes microglial proliferation via mTOR signaling and PLCG2 (phospholipase C gamma 2) activity. Importantly, ATV:TREM2 does not cause ERK1/2 phosphorylation nor pro-inflammatory signaling in microglia (van Lengerich et al., 2023). ATV:TREM2 also augmented phagocytosis and CSF1R (colony-stimulating factor 1 receptor) levels in human TREM2 transgenic TfR^mu/hu^ mice (CSF1R is pivotal to the survival of microglia; Elmore et al., 2014; van Lengerich et al., 2023). Moreover, ATV:TREM2 significantly decreased triglyceride (lipid droplet components) accumulation (Olzmann and Carvalho, 2019; Andreone et al., 2020; van Lengerich et al., 2023) by inducing mitochondrial fatty acid oxidation and PLCG2-dependent respiration in microglia (Loving and Bruce, 2020; van Lengerich et al., 2023). In addition, in an AD mouse model, ATV:TREM2 boosted microglial activity and glucose metabolism (van Lengerich et al., 2023). This is of particular significance because glucose hypometabolism is associated with AD pathology (Reiman et al., 2003; Jack et al., 2013; van Lengerich et al., 2023).

In partnership with Takeda, Denali Therapeutics has tested ATV:TREM2 (named DNL919) in a phase 1 trial of 47 healthy subjects in the Netherlands that ended on June 8, 2023 (ClinicalTrials.gov, 2022b). On August 9, 2023, it was revealed that Takeda and Denali Therapeutics will no longer be moving forward with the development of ATV:TREM2 (DNL919) due to “safety signals of moderate, reversible hematologic effects” that were “observed at the highest dose tested, suggesting a narrow therapeutic window” for AD patients (Lynch, 2023). Instead, the companies will explore “back-up molecules in preclinical development” (Lynch, 2023).

AL002 is a humanized monoclonal anti-TREM2 antibody currently in a phase 2 clinical trial (ClinicalTrials.gov, 2020a). AL002 was advanced to clinical testing following overall promising preclinical findings. In primary myeloid cells, AL002 was shown to activate human TREM2 and improved cell survival (Wang et al., 2020; Ward et al., 2021). In terms of AL002’s derivatives, however, the results are inconsistent. Jain et al. (2022) administered stereotactic intracerebral injections of human AD-tau in 5xFAD mice to give rise to “amyloid-induced tau seeding and spreading” and found that AL002a increased the clustering of DAM state microglia around amyloid plaques, thereby confirming that AL002a increased TREM2 signaling in a murine model of AD. They also found that AL002a surprisingly increased NP-tau pathology seeding and spreading, peri-plaque NP-tau pathology, plaque-associated neuritic dystrophy, and loss of peri-plaque synaptic puncta. Additionally, they found that AL002a did not impact amyloid plaque burden nor fibrillar plaque conformation in the mice, nor AD-tau phagocytosis and degradation in bone marrow-derived macrophages (Jain et al., 2022). Conversely, Price et al. (2020) administered intracranial injections of AL002a in 5xFAD mice and found that amyloid-associated gene expression signature was reversed, microglia were recruited to amyloid plaques, less Aβ was deposited, and spatial learning and new object recognition improved. Wang et al. (2020) investigated the effects of AL002c, an agonistic TREM2 mouse antibody that binds human TREM2, on 5xFAD mice expressing either the human TREM2 common variant or the human TREM2 R47H variant. They found that intraperitoneal injections of AL002c induced metabolic activation of microglia and microglial proliferation, increased microglial surveillance and Aβ phagocytosis, decreased soluble Aβ levels, and decreased filamentous plaques and neurite dystrophy. However, they found that AL002c did not have an effect on total amyloid plaque load nor overall plaque size, and that AL002c did not cause microglia to cluster around amyloid plaques more (Wang et al., 2020).

Overall, the preclinical data regarding AL002 are mixed. These inconsistencies could be due to the variety of animal models used, e.g., tau seeding, 5xFAD, and TREM R47H. Since recent data investigating the role of TREM2 have shown that AD pathology may be dependent upon the presence of humanized genes in transgenic mouse models, namely *APOE4* (Gratuze et al., 2022), the mouse model is critical in studies investigating TREM2 function and agonism. Differential epitope specificity of the AL002 variants may also contribute to the variable downstream effects. Small differences in epitope binding could have a marked effect on TREM2-mediated downstream signaling, microglial activation, and effector functions of microglia. Since TREM2 binds a variety of ligands, it is also plausible that anti-TREM2 mAb binding may alter the endogenous interactions between TREM2 and its binding partners. Although these findings raise the question of whether TREM2 agonism or antagonism may be more beneficial in the context of AD, they also highlight the need for basic studies that interrogate the ligand-specific activation of TREM2-mediated signaling.

Despite inconsistencies in preclinical data, AL002 was advanced to a phase 1 INVOKE trial (ClinicalTrials.gov, 2018) that was completed in November 2020, which gauged the safety, immunogenicity, and tolerability of AL002 in 69 healthy human adults and adults with mild-to-moderate AD. The single ascending dose (SAD) phase of the study consisted of healthy adult participants that received a single dose of AL002 ranging from 0.003 to 60 mg/kg or a similar dose of placebo (saline) via an IV infusion (Ward et al., 2021). Subjects were followed for 12 weeks following their single AL002 dose or placebo dose. CSF concentrations of the following TREM2 signaling biomarkers were measured: sTREM2 (soluble TREM2), sCSF1R (soluble colony stimulating factor 1 receptor), SPP1 (secreted phosphoprotein 1), and IL1RN (interleukin-1 receptor antagonist). SPP1 and IL1RN regulate neuroinflammation and SPP1 also promotes microglial survival (Yu et al., 2017; Heneka et al., 2018; Thome et al., 2020; Ward et al., 2021). 69.8% of AL002 subjects experienced adverse events compared to 81.8% of placebo subjects (Ward et al., 2021). Two subjects withdrew from AL002 treatment due to adverse events including mild nausea, moderate paresthesia, and mild retching. AL002 decreased sTREM2 in subject CSF in a dose-dependent fashion, showing successful target engagement. AL002’s activation of TREM2 causes TREM2 to internalize, thereby decreasing sTREM2 production at the microglial cell surface. AL002 also elevated CSF levels of sCSF1R, SPP1, and IL1RN (Ward et al., 2021), suggesting that AL002 can modulate microglial metabolism and function in the human brain.

**Figure 3:**
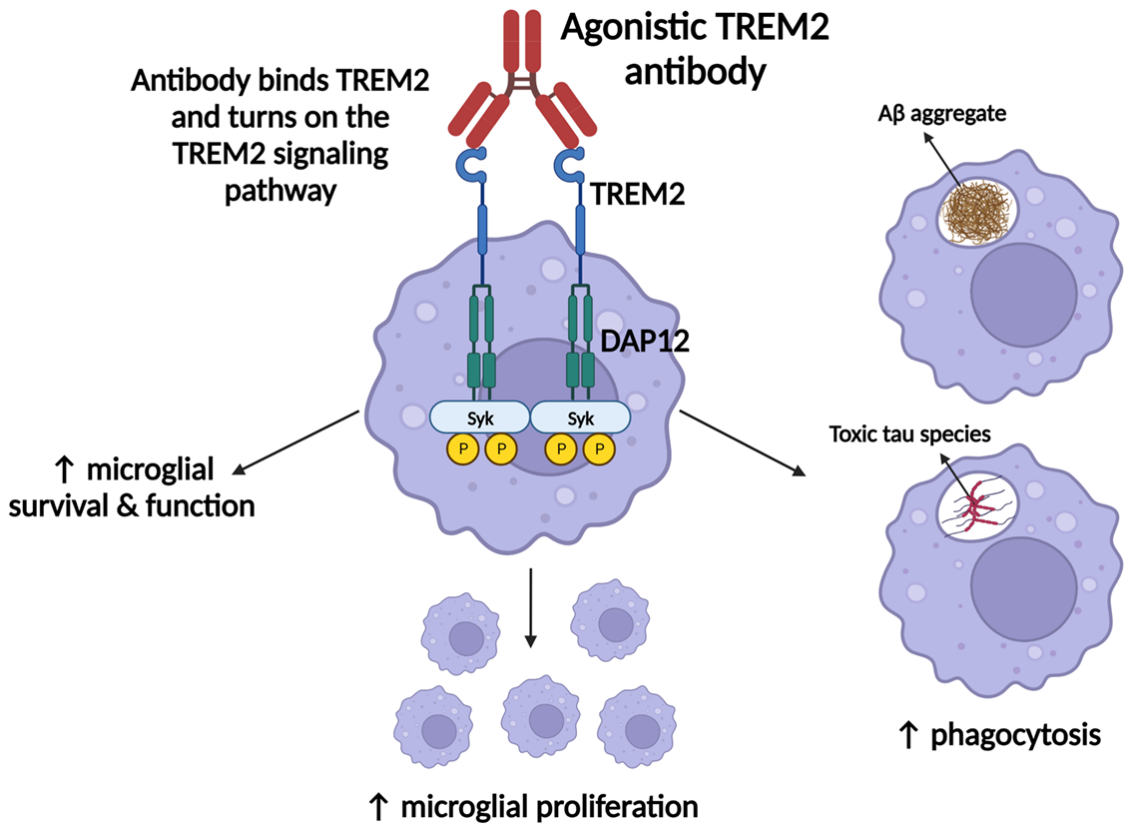
ATV:TREM2 and AL002’s mechanism of action. Once each agonistic TREM2 antibody binds TREM2, the TREM2 signaling pathway is activated, which promotes microglial survival, function, proliferation, and phagocytosis of AD pathologies. Figure adapted from Alector, Inc (2021) and Shugart (2020). Information derived from Ward et al. (2021) and Shugart (2020). Created with BioRender.com.

AL002 has since been advanced to a phase 2 clinical trial (INVOKE-2) to test efficacy and safety in an estimated 328 early AD subjects, and is projected to end in January of 2024 (ClinicalTrials.gov, 2020a; Ward et al., 2021). Whereas ATV:TREM2 prevents TREM2 cleavage to promote signaling, AL002 binds TREM2 to enhance signaling (Figure 3; Shugart, 2020; Alector, Inc, 2021; Ward et al., 2021). Overall, AL002-mediated TREM2 signaling is proposed to enhance microglial survival, function, proliferation, and phagocytosis (Figure 3; Alector, Inc, 2021; Ward et al., 2021).

Since microglia are involved in many of the facets of AD pathogenesis, therapeutics that target TREM2 to restore microglial survival, metabolism, and function are a significant and promising step toward disease-modifying AD treatments. Indeed, since microglia regulate multiple pathways associated with AD neuropathogenesis, therapeutics that target microglia may also be beneficial at different stages of the disease, thereby having the potential to markedly improve outcomes in AD patients.

## 8. Sargramostim: pro-inflammatory cytokine with positive cognitive effects

Microglia also express the granulocyte-macrophage colony-stimulating factor receptor (GM-CSFR) and proliferate in a non-inflammatory fashion following GM-CSF binding (Suzumura et al., 1990; Dikmen et al., 2020). Sargramostim (brand name Leukine) is a recombinant human GM-CSF and is already an FDA-approved treatment for bone marrow stimulation (Potter et al., 2021). Sargramostim/GM-CSF holds promise as an AD therapeutic due to its ability to increase microglial proliferation and boost microglial activity and cytokine production in such a way that demonstrates positive cognitive effects (Jim et al., 2012; Potter et al., 2021).

A pilot phase 2 study completed in December 2019 was conducted to evaluate the safety and efficacy of sargramostim in the treatment of 44 mild-to-moderate AD subjects (ClinicalTrials.gov, 2011). Dermatological and gastrointestinal adverse events as well as headaches occurred in some study participants (Potter et al., 2021; Mayo Clinic, 2023). However, serious adverse events as well as ARIA did not occur (Potter et al., 2021). At the end of treatment (after 3 weeks), sargramostim increased the numbers of monocytes (*p*-value: 0.0005), lymphocytes (*p*-value: 0.0512), and neutrophils (*p*-value < 0.0001), and the cytokines IL-2 (*p*-value: 0.0022), IL-6 (*p*-value: 0.0154), IL-10 (*p*-value: 0.0003), and TNF-α (*p*-value < 0.0001) in the blood. IL-8 levels (*p*-value: 0.0052) and the albumin/globulin ratio (*p*-value: 0.0029) decreased. Mean plasma Aβ40 levels in sargramostim subjects increased 8.4% from baseline (*p*-value: 0.0127) and were 10% greater than those of placebo (saline) subjects (*p*-value: 0.0105; Potter et al., 2021). Since mean plasma Aβ40 levels typically decrease in AD, the results suggest that there is a decreased uptake of monomeric and oligomeric Aβ in the brain (Janelidze et al., 2016; Nakamura et al., 2018; Potter et al., 2021). Moreover, sargramostim subjects had a 17% decrease in total tau plasma levels compared to baseline (*p*-value: 0.0327) and a 24% decrease compared to the baseline change of placebo subjects (*p*-value: 0.0174; Potter et al., 2021). Also, sargramostim subjects experienced a 40% decrease in UCH-L1 (ubiquitin carboxyl-terminal hydrolase L1) plasma levels compared to baseline (*p*-value: 0.0017) and a 42% decrease compared to placebo subjects (*p*-value: 0.0019; Potter et al., 2021). UCH-L1 is secreted by injured neurons into the CSF and blood, so a reduction in UCH-L1 is indicative of neuroprotection (Quanterix, 2020). However, amyloid load, measured using Amyvid (the first FDA-approved imaging agent for estimating amyloid plaque density in the brain; Rabinovici et al., 2007; Food and Drug Administration, 2012), did not change with sargramostim treatment (Potter et al., 2021).

Most AD drugs are specific in that they have a certain target in the AD pathway, but sargramostim simultaneously targets hematopoiesis and the innate immune system as a whole (Potter et al., 2021). Since NSAID clinical trials have failed at treating AD, boosting (rather than mitigating) inflammation may be a suitable alternative strategy (Breitner et al., 2014; Potter et al., 2021). Inflammation may be seen as a double-edged sword. On one hand, it serves a protective role as it protects the brain from “infection, toxins, and injury,” but once there is an imbalance between pro- and anti-inflammatory signaling in the brain, chronic inflammation results (Meraz-Ríos et al., 2013; Kinney et al., 2018). Furthermore, sargramostim treatment significantly increased IL-6 levels (among other cytokines), which might be a cause for concern. However, sargramostim may be a successful paradoxical therapeutic, i.e., perhaps inducing inflammation can boost protective mechanisms to improve outcomes in AD. Sargramostim is currently in a phase 2 trial to gauge its safety and efficacy in an estimated 42 mild-to-moderate AD subjects, and is set to end in July of 2024 (ClinicalTrials.gov, 2021b).

## 9. CMS121: small molecule fatty acid synthase inhibitor

As detailed earlier, disordered lipid metabolism and transport have been increasingly implicated in AD neuropathogenesis, thereby promoting the search for lipid-targeting therapeutics. Specifically, in AD, reactive oxygen species (ROS) peroxidize lipids, particularly PUFAs (polyunsaturated fatty acids), leading to the formation of toxic lipid peroxidation byproducts that act as electrophilic aldehydes that crosslink with DNA and/or covalently bind amino acids (Huang et al., 2016; Gaschler and Stockwell, 2017; Ates et al., 2020). Lipid peroxidation byproducts also induce inflammation (Yadav and Ramana, 2013; Ates et al., 2020). Several studies have shown increased lipid peroxidation in AD patients’ brains (Sultana et al., 2006; Bradley-Whitman and Lovell, 2015; Ates et al., 2020). Failure to control lipid peroxidation leads to ferroptosis and cell death (Dixon et al., 2012; Ralhan et al., 2021). In an attempt to prevent lipid peroxidation, fatty acids on cell membranes are converted into triglycerides and sequestered into LDs (lipid droplets; Bailey et al., 2015; Liu et al., 2015; Ralhan et al., 2021). However, when these neutral lipid stores become overwhelmed, this can negatively affect cellular functions. For example, microglia that accumulate excessive LDs exhibit dysfunctional phagocytosis, produce excessive ROS, and secrete pro-inflammatory cytokines (Mosher and Wyss-Coray, 2014; Marschallinger et al., 2020). Since fatty acid synthase (FASN) is a rate-limiting enzyme in *de novo* lipogenesis, reducing fatty acid synthesis by inhibiting FASN is a promising strategy to mitigate lipid peroxidation in AD.

**Figure 4:**
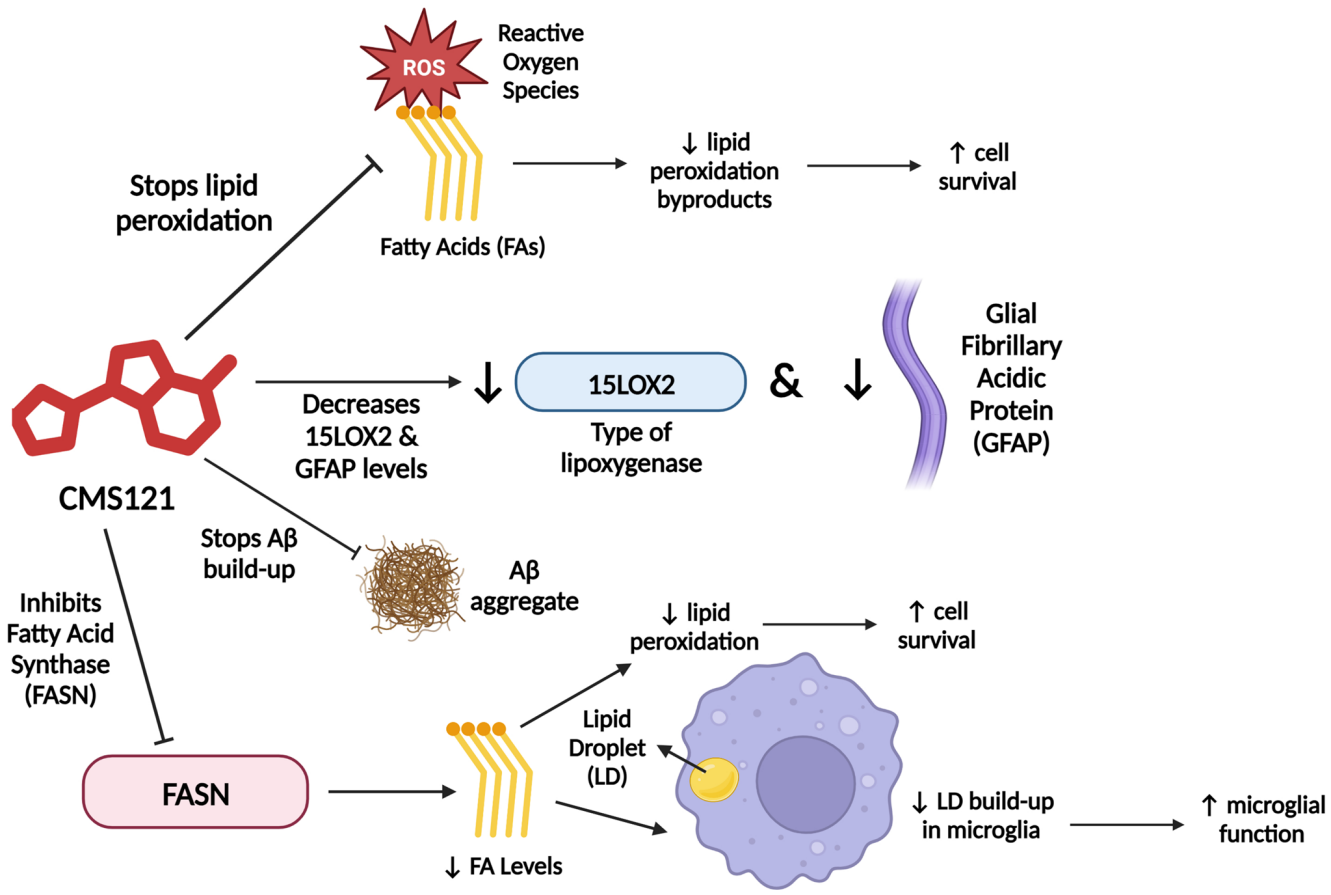
CMS121’s mechanism of action. CMS121 has a variety of functions. It stops ROS (Reactive Oxygen Species) from peroxidizing PUFAs (Polyunsaturated Fatty Acids), which increases cell survival, decreases levels of the inflammatory markers 15LOX2 (a type of LOX enzyme) and GFAP (Glial Fibrillary Acidic Protein), prevents Aβ accumulation and subsequent aggregate formation, and inhibits FASN (Fatty Acid Synthase), which decreases fatty acid levels, thereby decreasing lipid peroxidation and LD (lipid droplet) accumulation in microglia. Information derived from Ates et al. (2020). Created with BioRender.com.

The FASN inhibitor CMS121 is a small molecule derivative of the flavonoid fisetin, a dietary antioxidant with neurotrophic, anti-carcinogenic, and anti-inflammatory effects (Khan et al., 2013; Prior et al., 2014; Ates et al., 2020; Figure 4). CMS121 extricates cells from oxytosis, a type of regulated cell death caused by glutathione depletion, and ferroptosis, a non-apoptotic, iron-dependent form of regulated cell death (Prior et al., 2014; Lewerenz et al., 2018; Ates et al., 2020).

*In vitro* studies showed that CMS121 prevented RSL3 (RAS-selective lethal 3)-mediated lipid peroxidation in neuronal HT22 cells (*p*-value < 0.001; Ates et al., 2020). CMS121 also increased protection against oxytosis/ferroptosis in the presence of glutamate (*p*-value < 0.0001) and erastin (*p*-value < 0.01), but not RSL3 (glutamate, erastin, and RSL3 are oxytosis/ferroptosis inducers). CMS121 inhibited lipid peroxidation caused by bacterial lipopolysaccharide (LPS) in BV2 microglia-like cells (*p*-value < 0.001). CMS121 also lowered 4HNE (4-hydroxynonenal) levels, a cytotoxic byproduct and marker of lipid peroxidation (Ayala et al., 2014), in the hippocampi of AD mice (*p*-value < 0.01; Ates et al., 2020). CMS121 was also capable of reducing pro-inflammatory factors such as iNOS (*p*-value < 0.001), TNF-α (*p*-value < 0.0001), and COX2 (*p*-value < 0.05) following LPS treatment, recapitulating the effects of *FASN* knockdown. Furthermore, CMS121 reduced GFAP (glial fibrillary acidic protein) levels in the hippocampi of AD mice (*p*-value < 0.05). Since GFAP is a marker of reactive astrocytes, these data suggest that CMS121 can improve glial cell function *in vivo* (Oostveen et al., 1998; Ates et al., 2020).

Consistent with its function, CMS121 appeared to reduce lipid levels in an AD mouse model (Ates et al., 2020). Specifically, the levels of endocannabinoids (*p*-value: 0.048), fatty acids (*p*-value < 0.0001), and PUFAs (*p*-value < 0.0001) were significantly reduced in CMS121-treated AD mice. However, ceramide levels were significantly increased in AD mice following CMS121 treatment (*p*-value: 0.001; Ates et al., 2020). Since ceramides have been implicated in the neuronal cell death that causes AD (Filippov et al., 2012), this may raise some concerns. Indeed, AD patients have increased ceramide brain levels, which are highest in patients with multiple neuropathologic abnormalities (Filippov et al., 2012). Nonetheless, increased ceramide levels are consistent with FASN inhibition, where we would expect an increased pool of malonyl-CoA to inhibit CPT-1 (carnitine palmitoyltransferase 1)-mediated transport of fatty acids into the mitochondria for β-oxidation, thereby shunting fatty acyl-CoA towards ceramide synthesis. Thus, in clinical trials of CMS121, ceramide levels should be evaluated. Measures of peripheral ceramides such as ceramide CERT scores may have some utility (Hilvo et al., 2020).

Overall, CMS121 shows favorable effects *in vitro* and *in vivo*, but it remains to be seen how well it performs in human AD patients. A phase 1 study that evaluated the “safety, tolerability, and pharmacokinetics” of CMS121 was completed in 99 healthy subjects on December 17, 2022 (ClinicalTrials.gov, 2022d), but results are yet to become available. It is expected, however, that CMS121 will yield successful results in clinical trials.

## 10. Other therapeutics in clinical trials: bryostatin and benfotiamine

Although we have focused on several therapeutics in depth, many more are at different stages of development. For example, bryostatin is a marine macrocyclic lactone that activates the protein kinase C epsilon (PKCε) enzyme, and has been proven to augment synaptic numbers through synaptic growth factors like BDNF (brain-derived neurotrophic factor), NGF (nerve growth factor), and IGF-I (insulin-like growth factor I; Sun et al., 2009; Sen et al., 2012; Farlow et al., 2019). PKC turns on α-secretase as well as enzymes responsible for breaking down Aβ, stabilizes mRNAs of growth factors, and stops tau phosphorylation regulated by GSK-3β (Alkon et al., 2007; Farlow et al., 2019). Bryostatin is well-tolerated and significantly turns on PKCε at lower doses (less than 30 μg/m^2^/week), but downregulates or even stops the function of PKCε at higher doses (Farlow et al., 2019). Compassionate use trials showed that bryostatin improves prognoses in patients with more advanced forms of AD (Nelson et al., 2017; Farlow et al., 2019). Synaptogenix’s phase 2 trial evaluated bryostatin’s long-term efficacy for moderately severe AD treatment (ClinicalTrials.gov, 2020c), and revealed that bryostatin did not meet the study’s primary endpoint: change from baseline in the Severe Impairment Battery (SIB) total score at week 28 when the second course of treatment was completed (Priyan, 2022).

Benfotiamine is perhaps a more promising therapeutic. Preclinical models have shown the synthetic vitamin B1 precursor to be an all-rounder that improves AD pathologies like plaques (Pan et al., 2010; Sambon et al., 2019), NFTs (Pan et al., 2010; Tapias et al., 2018), decreased glucose metabolism (Thornalley et al., 2007), oxidative stress (Balakumar et al., 2010; Raj et al., 2018), increased advanced glycation end product (AGE) levels (Peyroux and Sternberg, 2006), inflammation (Balakumar et al., 2010; Raj et al., 2018), and cognitive impairment (Gibson et al., 2020). In a phase 2a clinical trial (ClinicalTrials.gov, 2014), ADAS-Cog score increase in the benfotiamine group was 43% lower than the placebo group, which translates to less cognitive decline in the benfotiamine group (*p*-value: 0.125; Gibson et al., 2020). CDR score worsening was 77% lower in the benfotiamine group compared to the placebo group (*p*-value: 0.034), and this effect was more potent in *APOE* ε4 non-carriers. Benfotiamine decreased AGE increases (*p*-value: 0.044), and this effect was more potent in *APOE* ε4 non-carriers. Impressively, benfotiamine’s effects were present after a year (*p*-value: 0.002; Gibson et al., 2020). A “follow-on” phase 2 trial will be conducted to evaluate benfotiamine’s effects in early AD subjects for 18 months (trial not yet listed in a registry; AlzForum, 2022).

The aforementioned therapeutics are not the only ones in clinical trials for AD treatment. Trials currently in-progress and a list of those funded by the National Institute on Aging (NIA) can be found at this website: https://www.nia.nih.gov/research/ongoing-AD-trials. Table 1 lists the therapeutics discussed in this review and their pertaining information.

**Table 1:** Therapeutics and Pertaining Information.

Therapeutic	Description	Administration route and side effects	FDA Approved?	Latest ClinicalTrial.gov title and identifier	Latest clinical trial phase
Aducanumab	Anti-amyloid mAb	Intravenous (IV) infusion Blurry vision Vision changes Confusion Dizziness Falls Hallucinations Headache Holding false beliefs that cannot be changed by fact Depression Anxiety Nausea Nightmares or unusually vivid dreams Movement, walking, or speech problems Seizures Sleepiness or unusual drowsiness Unusual excitement, nervousness, or restlessness Vomiting Diarrhea Brain swelling (ARIA-E) Brain bleeding (ARIA-H) Infusion reactions/hypersensitivity	Yes	A study to evaluate safety and tolerability of Aducanumab in participants with Alzheimer’s disease who had previously participated in the Aducanumab Studies 221 AD103, 221 AD301, 221 AD302 and 221 AD205: NCT04241068 A study to verify the clinical benefit of Aducanumab in participants with early Alzheimer’s disease (ENVISION): NCT05310071	Phase 3b (ends February 3, 2025) Phase 3b/4 (ends October 31, 2026)
Lecanemab	Anti-amyloid mAb	Intravenous (IV) infusion Back pain Blurry vision Vision changes Tightness in chest Chills Confusion Diarrhea Dizziness Faintness Lightheadedness when getting up suddenly from lying down or sitting Drowsiness Feeling shaky Fever Flushing General malaise Headache Joint pain Appetite loss Muscle aches Muscle pain Nausea Vomiting Nervousness Pale skin Pounding in the ears Runny nose Seizures Shivering Bradycardia or tachycardia Sore throat Sweating Breathing problems Sleeping problems Unusual tiredness or weakness Cough or hoarseness Lower back or side pain Painful or difficult urination Brain swelling (ARIA-E) Brain bleeding (ARIA-H) Infusion reactions/hypersensitivity	Yes	A study to evaluate safety, tolerability, and efficacy of Lecanemab in subjects with early Alzheimer’s disease: NCT01767311 A study to confirm safety and efficacy of Lecanemab in participants with early Alzheimer’s disease (Clarity AD): NCT03887455 AHEAD 3–45 Study: a study to evaluate efficacy and safety of treatment with Lecanemab in participants with preclinical Alzheimer’s disease and elevated amyloid and also in participants with early preclinical Alzheimer’s disease and intermediate amyloid: NCT04468659	Phase 2 (ends February 20, 2025) Phase 3 (ends September 15, 2027) Phase 3 (ends October 25, 2027)
Donanemab	Anti-amyloid mAb	Intravenous (IV) infusion As shown in AD clinical trials so far: Nausea Urinary tract infections Diarrhea Vomiting Anxiety Brain swelling (ARIA-E) Brain bleeding (ARIA-H) Infusion reactions/hypersensitivity *Donanemab is expected to have higher risks than lecanemab and aducanumab*	Not yet (should be approved by the end of 2023)	A follow-on study of Donanemab (LY3002813) with video assessments in participants with Alzheimer’s disease (TRAILBLAZER-EXT): NCT04640077 A study of Donanemab (LY3002813) compared with Aducanumab in participants with early symptomatic Alzheimer’s disease (TRAILBLAZER-ALZ 4): NCT05108922 A study of different Donanemab (LY3002813) dosing regimens in adults with early Alzheimer’s disease (TRAILBLAZER-ALZ 6): NCT05738486 A study of Donanemab (LY3002813) in participants with early Alzheimer’s disease (TRAILBLAZER-ALZ 2): NCT04437511 A study of Donanemab (LY3002813) in participants with early symptomatic Alzheimer’s disease (TRAILBLAZER-ALZ 5): NCT05508789 A Donanemab (LY3002813) prevention study in participants with Alzheimer’s disease (TRAILBLAZER-ALZ 3): NCT05026866	Phase 2 (ends March 5, 2024) Phase 3 (ends July 2, 2024) Phase 3 (ends May 13, 2025) Phase 3 (ends August 22, 2025) Phase 3 (ends June 11, 2027) Phase 3 (ends November 8, 2027)
CT1812	Sigma-2 receptor antagonist	Oral administration As shown in AD clinical trials so far: Nausea Vomiting Headache Fatigue Lethargy Gastrointestinal disturbances Lymphocytopenia	Not yet (still in clinical trials)	Study to evaluate the safety, tolerability and efficacy of CT1812 in subjects with mild to moderate dementia with Lewy bodies (COG1201): NCT05225415 A study to evaluate the safety and efficacy of CT1812 in subjects with mild to moderate Alzheimer’s disease: NCT03507790 A Study to evaluate the safety and efficacy of CT1812 in early Alzheimer’s disease: NCT05531656	Phase 2 (ends April 15, 2024) Phase 2 (ends July 26, 2024) Phase 2 (ends August 2026)
Sargramostim	Granulocyte-macrophage colony-stimulating factor	Subcutaneous (SC) injection As shown in AD clinical trials so far: Dermatological side effects Gastrointestinal disturbances Headache	Not approved yet for use in AD (still in clinical trials)	Phase II trial to evaluate safety and efficacy of GM-CSF/Sargramostim in Alzheimer’s disease (SESAD): NCT04902703	Phase 2 (ends July 2024)
ATV:TREM2 (DNL919)	Agonistic TREM2 antibody	Intravenous (IV) infusion As shown in AD clinical trials so far: Moderate, reversible hematologic effects at the highest (undisclosed) dose	*No longer in development*	A study to evaluate the safety, tolerability, pharmacokinetics, and pharmacodynamics of DNL919 in healthy participants: NCT05450549	Phase 1 (ended June 8, 2023) *No longer in development*
AL002	Agonistic TREM2 antibody	Intravenous (IV) infusion As shown in AD clinical trials so far: ARIA in *APOE* ε4 homozygotes only	Not yet (still in clinical trials)	A Phase 2 study to evaluate efficacy and safety of AL002 in participants with early Alzheimer’s disease (INVOKE-2): NCT04592874 A long-term extension study to evaluate safety, tolerability, and efficacy of AL002 in Alzheimer’s disease: NCT05744401	Phase 2 (ends January 2024) Phase 2 (ends December 2025)
CMS121	Fatty acid synthase inhibitor	Oral administration Unknown side effects (data yet to be released)	Not yet (still in clinical trials)	Safety, tolerability and pharmacokinetics of CMS121, a drug candidate for Alzheimer’s disease, in healthy subjects (CMS121): NCT05318040	Phase 1 (ended December 17, 2022)
Bryostatin	Protein kinase C epsilon activator	Oral administration No side effects compared to placebo in AD clinical trials so far	Not approved yet for use in AD (still in clinical trials)	Bryostatin treatment of moderately severe Alzheimer’s disease: NCT04538066	Phase 2 (ended November 16, 2022)
Benfotiamine	Vitamin B1 precursor	Oral administration As shown in AD clinical trials so far: Increased liver enzyme count Increased urinary white blood cell count	Not approved yet for use in AD (still in clinical trials)	Benfotiamine in Alzheimer’s disease: a pilot study (Benfotiamine): NCT02292238	Phase 2 (ended September 8, 2020)

## 11. Non-pharmacological AD interventions

Disease-modifying pharmacological interventions are emerging and promise to be available to patients in the not too distant future. However, even if they exceed expectations, non-pharmacological interventions should also be utilized. Research favors the Mediterranean diet in the prevention of cognitive impairment and decline (Budson, 2020). This diet includes PUFA-rich foods such as fish, olive oil, avocados, and nuts, and fiber-rich fruits, vegetables, beans, and whole grains. Emphasis is especially placed on fish since it is the only food associated with a lower risk of cognitive decline (Budson, 2020). The MIND (Mediterranean-DASH Intervention for Neurodegenerative Delay) diet is another diet that AD patients can benefit from (Searor, 2022). This diet combines the Mediterranean diet and the DASH (Dietary Approaches to Stop Hypertension) diet to improve brain health and lessen cognitive decline and dementia. This diet is high in omega-3 fatty acids and includes leafy green vegetables, non-starchy vegetables, berries, nuts, beans, whole grains, fish, poultry, and olive oil (Searor, 2022). Furthermore, the ketogenic diet, ketone supplements, and dietary supplements such as medium-chain triglycerides could also benefit AD patients (Hersant and Grossberg, 2022). The ketogenic diet has been shown to improve cognition, memory, and quality of life in mild-to-severe AD patients (Tabaie et al., 2021). It is plausible that the aforementioned diets confer neuroprotection by metabolically reprogramming microglia in such a way that promotes beneficial microglial functions such as phagocytosis; these diets could also downregulate inflammation caused by microglia (Hornedo-Ortega et al., 2018; Augusto-Oliveira and Verkhratsky, 2021). It is also plausible that diets rich in shorter-chain fatty acids may be able to bypass and potentially restore the metabolic derangements in individuals carrying variants in genes regulating lipid and lipoprotein processing. However, due to inconsistencies in the field regarding the impact of diet on AD outcomes (Burckhardt et al., 2016), more work is needed in this area.

Exercise has been shown to be a particularly beneficial lifestyle intervention in the context of neurodegenerative disease. AD patients who engage in “long-term” exercise reap the benefits of better blood flow, greater hippocampal volume, and enhanced neurogenesis (Barnes, 2015; Meng et al., 2020). A lack of physical activity is “one of the most common preventable” AD risk factors and physical activity is associated with a lower risk of developing AD (Sattler et al., 2011; Meng et al., 2020). Mechanistically, exercise may mitigate AD risk by modulating microglial density, morphology, and phenotype (Augusto-Oliveira and Verkhratsky, 2021). In mice, exercise was shown to induce microglial proliferation in the superficial cortical layers and promote a ramified surveilling microglial state in the hippocampus (Ehninger, 2003; Olah et al., 2009; Augusto-Oliveira and Verkhratsky, 2021). In aged rats, exercise decreases the ratio between pro- and anti-inflammatory cytokines secreted by microglia in the hippocampus (Gomes da Silva et al., 2013; Barrientos et al., 2015; Augusto-Oliveira and Verkhratsky, 2021). Similarly, patients can also benefit from stress reduction. High stress levels worsen disease via Hypothalamic–Pituitary–Adrenal (HPA) axis activation resulting in increased levels of circulating corticosteroids (Selye, 1950; Justice, 2018). High stress levels accelerate AD pathogenesis and cognitive decline, and AD disrupts the neural circuitry involved in the stress response, thereby resulting in depression, anxiety, and aggressive behavior (Jeong et al., 2006; Sayer et al., 2008; Alexander et al., 2011; Carroll et al., 2011; Yaffe, 2012; Mah et al., 2015; Justice, 2018). It has been suggested that therapeutics that selectively decrease stress hormone levels like CRFR1 (corticotropin-releasing factor receptor-1) antagonists should be tested for efficacy in slowing AD progression (Campbell et al., 2015; Justice et al., 2015; Zhang et al., 2015, 2016; Justice, 2018). Overall, these findings suggest that diet and lifestyle modifications should be employed as preventative measures to reduce AD risk and should form a major part of the treatment plan for those diagnosed with AD and AD-related dementias.

## 12. Summary and future directions

**Figure 5:**
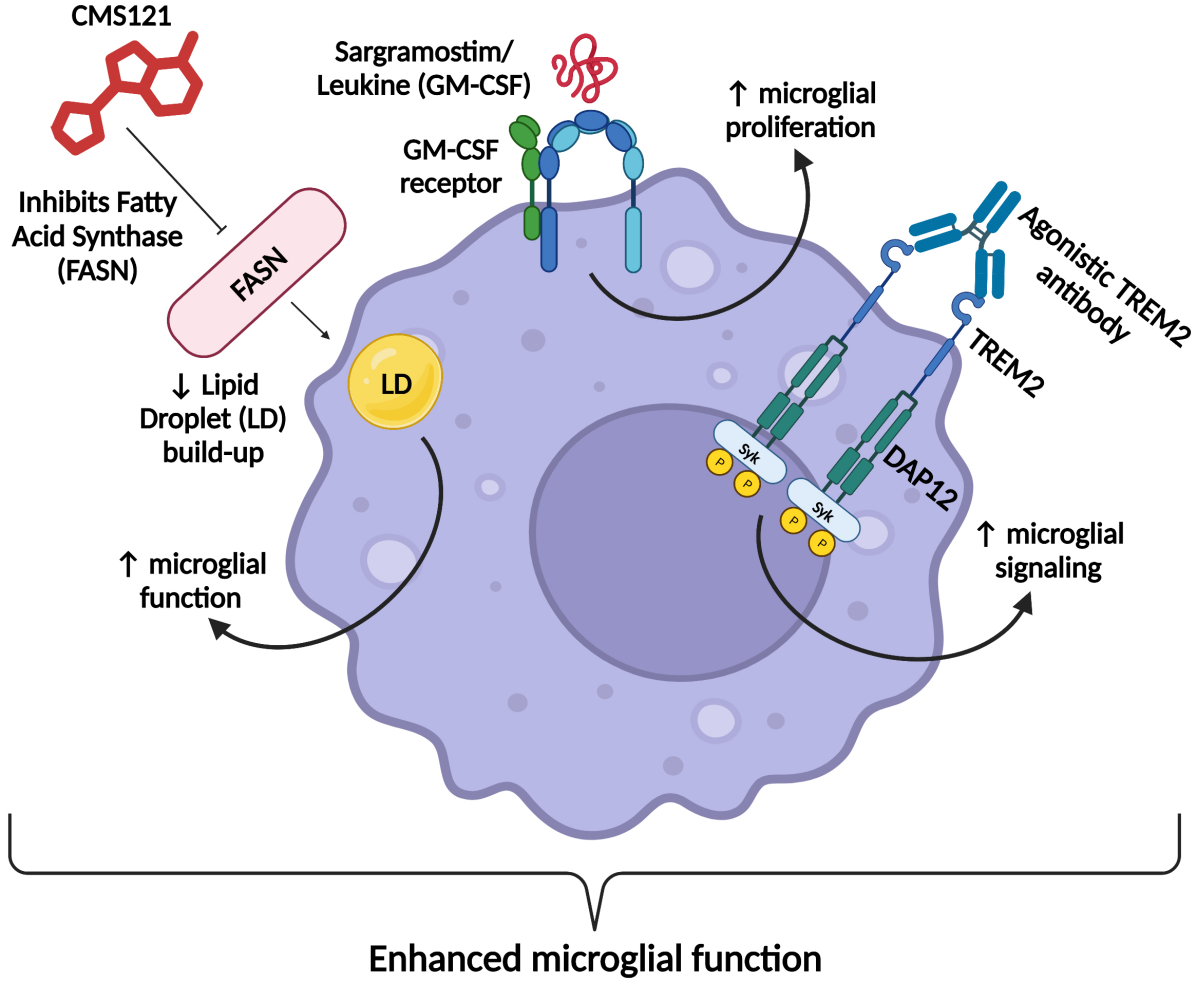
Summary figure. Agonistic TREM2 antibodies ATV:TREM2 and AL002 increase TREM2 signaling, sargramostim increases microglial proliferation, and CMS121 increases microglial function. These therapeutics collectively enhance microglial function and reduce AD pathology. Created with BioRender.com.

Standard-of-care AD therapeutics such as cholinesterase inhibitors and memantine only improve cognition for a few months before patients revert to their pre-medication cognitive state, and although anti-amyloid mAbs slow cognitive decline and disease progression, they are also associated with severe adverse effects. Nonetheless, anti-amyloid mAbs have set the stage for new AD treatments with true disease-modifying potential. Going forward, AD patients can expect many more therapeutics that will hopefully provide significant benefits and improve their prognoses. Notably, several of these newer approaches target lipid and lipoprotein processing and microglial function. For example, CT1812, the first σ2R antagonist to reach clinical trials, prevents Aβ oligomers from binding to lipoprotein receptor binding sites and promotes clearance of highly neurotoxic Aβ via the CSF to enhance synaptic function and cognition. Sargramostim, a recombinant human GM-CSF, binds to GM-CSFRs on microglia, leading to microglial proliferation and activation. Sargramostim’s overall pro-inflammatory nature might make it AD’s first paradoxical treatment. Furthermore, AL002 is an agonistic TREM2 antibody that binds TREM2 to enhance TREM2-mediated signaling and microglial proliferation and phagocytosis. CMS121 is an exciting and mechanistically distinct therapeutic. It stops lipid peroxidation, prevents the excessive formation of LDs in glial cells to increase cell survival and function, decreases oxidative stress and inflammatory markers, and prevents Aβ accumulation. Overall, these next-generation AD drugs work in a distinct but complementary fashion to improve microglial function and reduce AD pathology (Figure 5).

Since AD is a multifactorial disease, its treatment will require different therapeutics targeted at each AD pathology. Genetic testing will facilitate the identification of individuals who are at risk of developing exacerbated specific AD factors. For example, readily available screening for AD risk variants such as *APOE4*, may help guide clinical treatment. ADmark® Early Onset Alzheimer’s Evaluation developed by Athena Diagnostics, which detects sequence variants in the *PSEN1*, *PSEN2*, and *APP* genes and duplications in the *APP* gene from a patient’s whole blood sample using next-generation sequencing and dosage analysis (Athena Diagnostics, 2023), is also another step towards precision medicine.

In addition to genetic screening, biomarker identification brings us one step closer to precision medicine approaches for AD patients and at-risk individuals. So far, several AD biomarkers have been identified such as Aβ42, phosphorylated tau, and total tau CSF levels (Gunes et al., 2022). Unfortunately, CSF biomarker testing is quite invasive and physically burdens the patient, thereby highlighting the need for more biomarkers in different biofluids with testing methods that are as minimally invasive as possible. However, promising findings from Huan et al. (2018) have identified new AD biomarkers (glucosylgalactosyl, hydroxylysine-H_2_O, and glutamine-carnitine) in saliva that could distinguish between AD patients, pre-symptomatic patients, and MCI patients. Recent advancements in artificial intelligence (AI) are also being used to diagnose AD, including techniques like retina and iris readings, electroencephalogram tests, and AI-based online language skills and memory tests (Czakó et al., 2020; Eyigoz et al., 2020; Meghdadi et al., 2021; Gunes et al., 2022). Gunes et al. (2022) also highlight new AD biomarkers including optical coherence tomography and optical coherence tomography angiography for measuring any abnormalities in eye vasculature. Overall, identifying individuals at risk of AD early on would allow individuals to implement lifestyle and dietary modifications that could help prevent AD neuropathogenesis. At-risk individuals could also undergo more frequent screening so that therapeutic interventions could be implemented in more favorable therapeutic windows. Although new therapeutic strategies are on the horizon, more investment in research that advances our mechanistic understanding of AD, alternative approaches to treating AD, and the identification of individuals with or at risk of AD are very much warranted.

## Author contributions

NT: Conceptualization, Validation, Writing – original draft, Writing – review & editing. KB: Conceptualization, Validation, Writing – original draft, Writing – review & editing, Funding acquisition, Project administration, Visualization.
